# Supplementary figures and images for: Is perfectionism associated with academic burnout through repetitive negative thinking?
Source: PeerJ. 2018 Jun 18;6:e5004. doi: 10.7717/peerj.5004 (PMC6011823; doi:10.7717/peerj.5004)

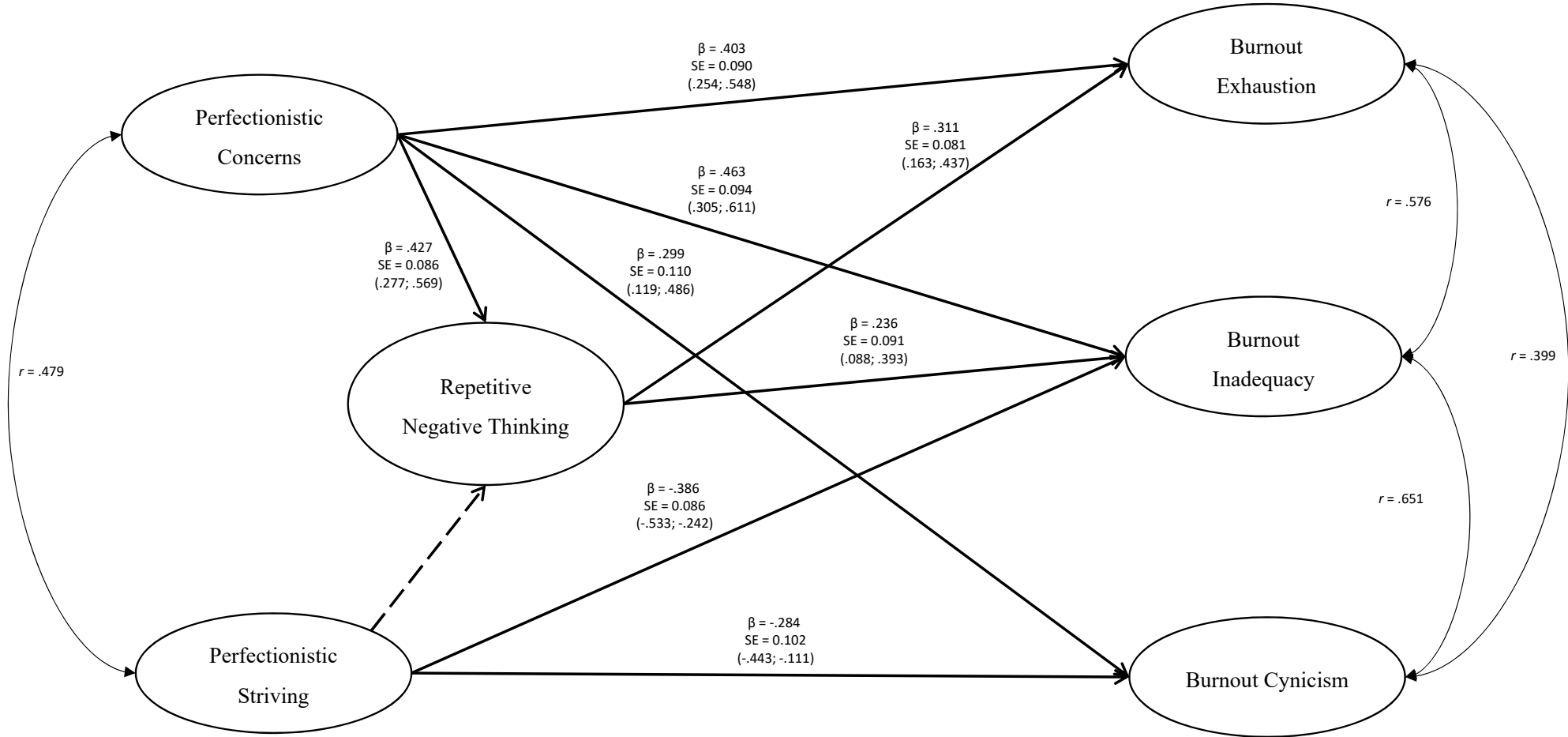

Supplement: Figure S1 — Initial tested model with all pathways between perfectionism, burnout, and repetitive negative thinking. Continuous arrows indicate significant pathways, whereas discontinuous arrows indicate non-significant pathways. Only significant pathways coefficients represented. All coefficients are standardized with 95% confidence intervals in brackets. [file peerj-06-5004-s002.pdf]
