# Supplementary material for: Is perfectionism associated with academic burnout through repetitive negative thinking?
Source: PeerJ. 2018 Jun 18;6:e5004. doi: 10.7717/peerj.5004 (PMC6011823; doi:10.7717/peerj.5004)
Supplement: Supplemental Information 2 — Questionnaire used in the present study presented on separate pages. [file peerj-06-5004-s003.pdf]

Demographics Questions

What is your age? __________ years

What is your gender? ☐ Male ☐ Female ☐ Unspecified

What is your current university enrolment? ☐ Full-time ☐ Part-time

How many years have you attended university? __________ years

Frost Multidimensional Perfectionism Scale

Please choose the option on the continuum that is most true for you.

Strongly Disagree	•	•	•	Strongly Agree
(1)	(2)	(3)	(4)	(5)

1. My parents set very high standards for me
2. Organisation is very important to me
3. As a child, I was punished for doing things less than perfect
4. If I do not set the highest standards for myself, I am likely to end up a second rate person
5. My parents never tried to understand my mistakes
6. It is important to me that I be thoroughly competent in everything I do
7. I am a neat person
8. I try to be an organised person
9. If I fail at university, I am a failure as a person
10. I should be upset if I make a mistake
11. My parents wanted me to be the best at everything
12. I set higher goals for myself than most people
13. If someone does a task at university better than me, then I feel like I failed the whole task
14. If I failed partly, it is as bad as a complete failure
15. Only outstanding performance is good enough in my family
16. I am very good at focusing my efforts on attaining a goal
17. Even when I do something very carefully, I often feel that it is not quite done right

18. I hate being less than the best at things

19. I have extremely high goals

20. My parents have expected excellence from me

21. People will probably think less of me if I make a mistake

22. I never felt like I could meet parent’s expectations

23. If I do not do as well as other people, it means I am an inferior human being

24. Other people seem to accept lower standards from themselves than I do

25. If I do not do as well all the time, people will not respect me

26. My parents have always had higher expectations for my future than I have

27. I try to be a neat person

28. I usually have doubts about the simple everyday things I do

29. Neatness is very important to me

30. I expect higher performance in my daily tasks than most people

31. I am an organised person

32. I tend to get behind in my work because I repeat things over and over

33. It takes me a long time to do something “right”

34. The fewer mistakes I make, the more people will like me

35. I never felt like I could meet parent’s standards

Repetitive Negative Thinking – 10

Please consider how you respond when you feel distressed or upset and circle the corresponding number that best reflects your experience WHEN YOU ARE DISTRESSED OR UPSET. Please be sure to read each statement carefully.

Not true at all	•	Somewhat true	•	Very true
1	2	3	4	5

When you are distressed or upset…

1. You have thoughts or images about all your shortcomings, failings, faults, mistakes.
2. You have thoughts or images about events that come into your head even when you do not wish to think about them again
3. You have thoughts or images that "*I won't be able to do my work because I feel so badly.*"
4. You have thoughts or images that are difficult to forget.
5. Once you start thinking about the situation, you can't stop.
6. You notice that you think about the situation a lot.
7. You have thoughts or images of the situation that you try to resist thinking about.
8. You think about the situation all the time.
9. You know you shouldn't think about the situation, but can't help it.
10. You have thoughts or images about the situation and wish it would go better.

School Burnout Inventory

Please choose the alternative that best describes your situation (estimation from previous month)

Completely disagree - Partly disagree - Disagree - Partly agree - Agree - Completely agree

1	2	3	4	5	6

I feel overwhelmed by my university work (EXH1)

I feel a lack of motivation in my university work and often think of giving up (CYN1)

I often have feelings of inadequacy in my university work (INAD1)

I often sleep badly because of matters related to my university work. (EXH2)

I feel that I am losing interest in my university work (CYN2)

I'm continually wondering whether my university work has any meaning (CYN3)

I brood over matters related to my university work a lot during my free time (EXH3)

I used to have higher expectations of my university work than I do now (INAD2)

The pressure of my university work causes me problems in my close relationships with others (EXH4)

Note. EXH = exhaustion at schoolwork; CYN = cynicism toward the meaning of school; INAD = sense of inadequacy at school.
